# Lime Stabilization of Tropical Soil for Resilient Pavements: Mechanical, Microscopic, and Mineralogical Characteristics

**DOI:** 10.3390/ma17194720

**Published:** 2024-09-26

**Authors:** Bruna Calabria Diniz, William Fedrigo, Thaís Radünz Kleinert, Giovanni dos Santos Batista, Washington Peres Núñez, Bethania Machado Correa, Lélio Antônio Teixeira Brito

**Affiliations:** 1 Postgraduate Program in Civil Engineering: Civil Construction and Infrastructure, Federal University of Rio Grande do Sul, Porto Alegre 90035-190, Brazil; bruna.diniz@ufrgs.br (B.C.D.); thais.kleinert@ufrgs.br (T.R.K.); washington.nunez@ufrgs.br (W.P.N.); bethania.correa@ufrgs.br (B.M.C.); lelio.brito@ufrgs.br (L.A.T.B.); 2 School of Technology, Pontifical Catholic University of Rio Grande do Sul, Porto Alegre 90619-900, Brazil; giovanni.batista@edu.pucrs.br

**Keywords:** soil-lime, flexural properties, microstructure, mineralogy, cementitious reactions

## Abstract

Lime stabilization is a sustainable technique due to its use of local materials, increased durability, reduced maintenance, and improved resistance to water action. This paper examines the impact of lime stabilization on the mechanical, microscopic, and mineralogical properties of a tropical soil. Two types of lime, calcitic and dolomitic, were tested at 3% and 5% by weight. Compressive, indirect tensile and flexural test results and statistical analysis revealed that calcitic lime mixtures had higher strength and stiffness, whereas dolomitic lime mixtures exhibited greater deformability with higher tensile strain at break. Scanning electron microscopy indicated that the soil’s porous matrix closed within 7 days for both lime types due to flocculation, with increased matrix interlocking over time. The calcitic lime mixture developed a more closed matrix compared to the dolomitic lime, which showed weaker cementing. X-ray diffraction analysis indicated higher consumption of clay minerals and a notable reduction in calcium hydroxide peaks in the lime-treated soils. The study concludes that calcitic lime provides better pavement performance for stabilizing the soil, enhancing its engineering properties while also being sustainable by reducing the need for raw material extraction and improving resilience to climate-related issues such as floods.

## 1. Introduction

The need for sustainable and economical pavements, which are less expensive and show better performance, has become a primary demand. Therefore, the use of lime for tropical soil modification and stabilization appears as a potential ally in these aspects, allowing the use of local soil as selected subgrade, subbase, and base pavement layers. The resultant material will present increased durability and high resistance to water action, reducing maintenance costs and improving resilience to climate-related issues such as floods. As an example, the study by Akula et al. [1] shows that soil from a canal treated with lime continues to gain strength even after five decades. Unlike pavements, such structures are directly and constantly exposed to water action, highlighting the importance of using lime for resilient infrastructure. Other studies have also reported that soils treated with lime can maintain their stiffness under climate-related conditions (such as freeze/thaw cycles and soaking) that untreated soils cannot [2].

Lime addition to soil increases the pH to a value favorable to the dissolution of silica and alumina in the clay, increasing cementing product formation [3]. In general, pozzolanic reactions improve the strength and stability of soil-lime mixtures. This happens due to the interaction between lime (rich in calcium) and clay (rich in silicates and aluminates) [4,5].

Regarding the different types of lime, calcitic lime has a calcium oxide (CaO) content between 90 and 100%, while dolomitic lime has approximately 60%. Dolomitic lime also has high levels of MgO (magnesium oxide)—about 40% [6]. According to the explanations by Bhattacharja et al. [7], in the presence of magnesium, as is the case with dolomitic lime, the available calcium per unit weight is reduced, which must be compensated by using a higher content. Furthermore, since magnesium hydroxide is significantly less soluble than calcium hydroxide, calcitic lime provides more free lime for stabilization [8].

Several studies have been conducted to analyze the advantages of soil stabilization with lime by evaluating its mechanical behavior using unconfined compressive strength (UCS) and indirect tensile strength (ITS) [9,10,11,12,13,14,15]. However, the strength and stiffness properties of chemically stabilized pavement layers, including soil-lime mixtures, are better characterized by flexural tests. Although the importance of such tests is acknowledged by well-known pavement design methods [16,17], the number of studies on this topic remains limited [18,19,20,21,22]. When it comes to soil-lime mixtures, the availability of flexural test results is even more limited [9,23].

To address this knowledge gap, the research reported here aimed to analyze the effects of curing time, lime type, and lime content on the mechanical behavior (especially flexural), as well as the microscopic and mineralogical characteristics of lime-stabilized tropical soil mixtures. Different curing times (7, 28, 90, 120, and 180 d), lime types (calcitic and dolomitic—CL and DL, respectively), and lime contents (3 and 5%) were considered. UCS, ITS, and flexural strength (FS) tests were carried out to evaluate the mixtures’ strength, stiffness, and deformability and obtain pavement design parameters. The results were also statistically evaluated. Scanning Electron Microscopy (SEM) and X-ray diffraction (XRD) analyses were conducted to understand the changes in the microstructural and mineralogical characteristics of the mixtures due to the cementing reactions.

## 2. Materials and Methods

**Figure 1:**
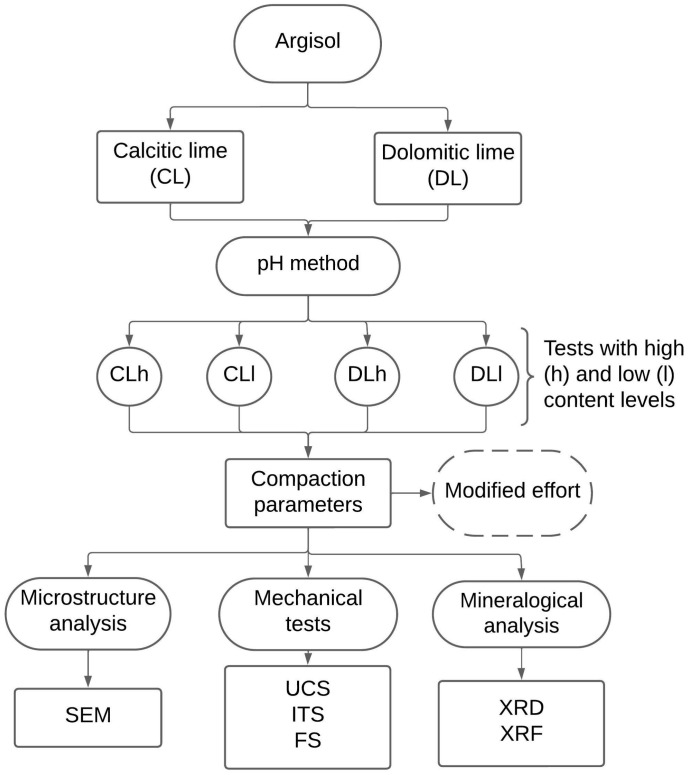
Methodological flowchart.

A methodological flowchart of the present study is presented in Figure 1.

The present study used a pure soil (PS) and two lime types (CL and DL) at two contents (3 and 5%). The soil was collected in Gravataí, Rio Grande do Sul, Brazil. It was characterized using X-ray diffraction (XRD), X-ray fluorescence (XRF) and chemical analyses. Besides, its basic geotechnical characterization (Atterberg limits and particle size distribution) was also carried out. The CL is from the state of Minas Gerais and it was classified as high-calcium hydrated. The DL is from the state of Rio Grande do Sul and it was characterized as special hydrated lime CH-II. Their physical and chemical characteristics were also determined.

The mixtures were designed using the pH method (initial consumption of lime, ICL) as per DNIT 419 [24] and ASTM D6276 [25]. UCS and ITS samples were compacted using modified effort (Modified AASHTO) and optimum moisture content, sealed in plastic bags, and cured in an environment with humidity > 95% and a temperature of 23 ± 2 °C. Based on the design method, two lime contents were defined, which were considered low and high values. UCS tests were performed in triplicate with 50 × 100 mm cylindrical samples as per ASTM D5102 [26]. For the ITS tests, loading strips were used [12] to distribute the stresses in the samples. ITS values were determined as per NBR 7222 [27].

The flexural test was based on D1635 [28] for soil-cement mixtures and on report 789 [29] of the National Cooperative Highway Research Program (NCHRP) for stabilized materials. Flexural tensile strength (FS), flexural tensile strain at break, and flexural modulus were computed. As per the mentioned methods, the curing time was 28 days. The selected lime content was the highest, since it would be expected to obtain high tensile strength values, leading to high-performance chemically stabilized pavement layers. Besides, the curing conditions were the same as those used for the UCS and ITS samples.

The microstructure analysis was performed using samples extracted from the specimens tested for UCS, with approximately 1 cm^2^, as planar as possible. After sample extraction, they were dried in an oven at 110 °C until reaching mass constancy. Subsequently, they were placed in plastic bags with silica gel sachets to avoid moisture gain. Then, the metallization process was carried out in vacuum equipment (Quorum Q150R ES/Plus, San Jose, CA, USA). A thin gold film was electrodeposited to facilitate the conduction of electrons by the SEM. The SEM analyses were carried out in an FEI Inspect F50 model (Hillsboro, OR, USA) and aimed to observe the textural changes that occurred due to adding lime to the soil at a microscopic level. SEM analyses were performed with soil and soil-lime samples (5% lime) cured for 7, 90, 120, and 180 d.

For XRD analyses, the samples from UCS were ground using a mortar and pestle. The experiments were carried out in an Empyrean Diffractometer, from Malvern Panalytical. A Cu Kα radiation source (40 kV and 30 mA), with a scan interval of 5–75° 2θ, and a step of 0.02°/1 s, was used. XRD analyses were conducted in soil-lime samples (5% lime) cured for 7, 28, 120, and 180 d.

## 3. Results

### 3.1. Soil and Lime Characterization

**Figure 2:**
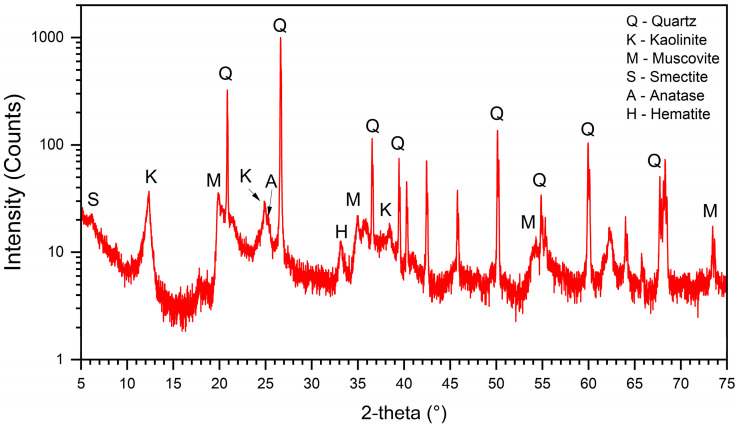
XRD diffractogram of PS.

**Figure 3:**
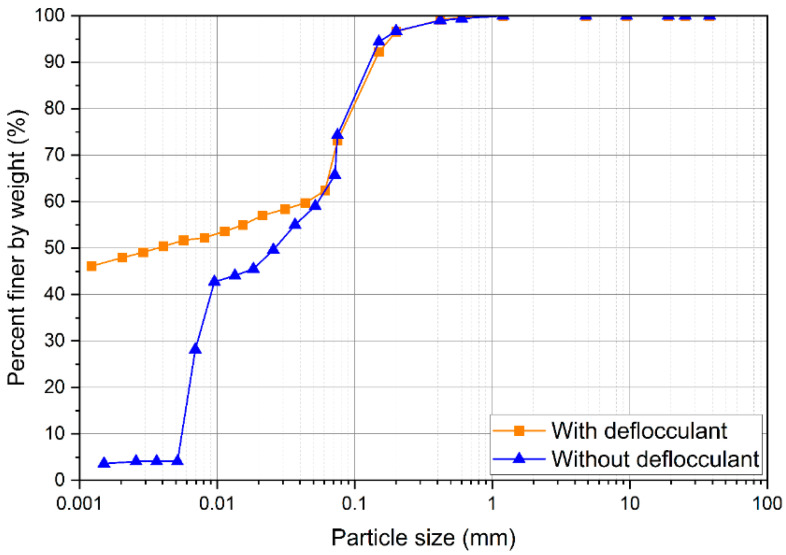
Particle size distribution curve of PS.

The pure soil XRD and XRF analyses are presented in Figure 2 and Table 1, respectively. The soil presented a high clay content (>60%) of low reactivity and an acid pH (4.4). Moreover, the exchangeable values of aluminum (Al), calcium (Ca), and magnesium (Mg) are classified as high, low, and medium, respectively. A high content of SiO_2_ (quartz) was observed through XRF analysis, in agreement with the XRD analysis. The cation exchange capacity (CEC) value of 14.2 cmolc/dm^3^ is classified as medium, standardized by the Brazilian Agricultural Research Corporation (EMBRAPA). Also, according to EMBRAPA, the soil was classified pedologically as a Typical Aluminum Red Argisol. The liquid limit and the plastic limit were 51 and 26, respectively, resulting in a plastic index of 25. Conforming to the American Association of State Highway and Transportation Officials (AASHTO), the soil is classified as A-7-6. Furthermore, according to the Unified Soil Classification System (USCS), the soil is a fine soil, denominated as CH (inorganic clay of high compressibility). The chemical analysis is presented in Table 2 and the particle size distribution is presented in Figure 3. The characterization of the limes is shown in Table 3.

### 3.2. Mix Design

**Figure 4:**
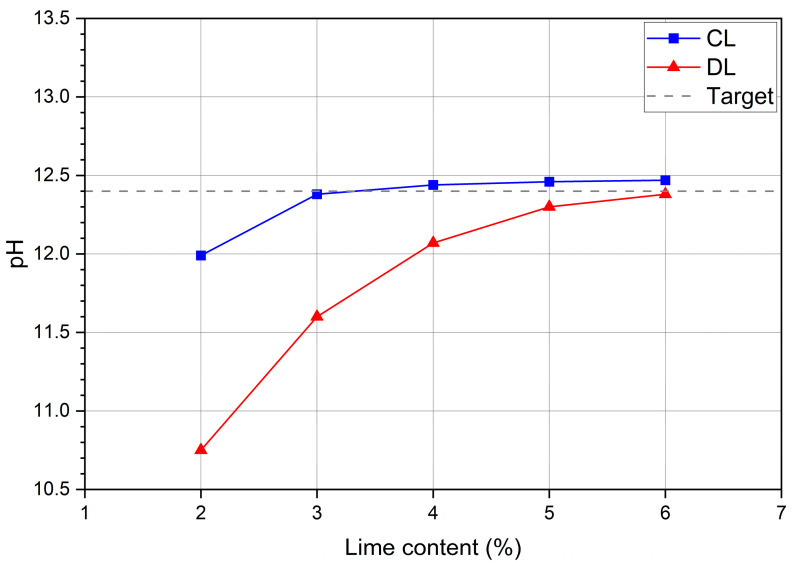
pH test results for soils samples with CL and DL.

Soil mixtures containing 2 to 6% CL and DL were used. As shown in Figure 4, CL presented better interaction with the clay minerals in the soil, revealing itself to be more reactive.

**Figure 5:**
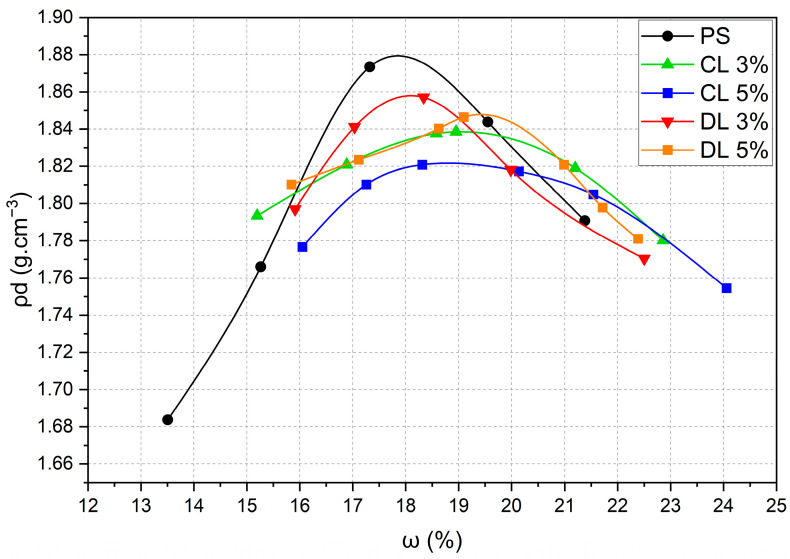
Compaction curves of the samples.

The target pH content estimated for stabilization purposes (12.4) was reached with 3% CL, remaining stable up to 6%. For DL, the target pH value was only achieved with 5%. Thus, it was decided to adopt lime contents of 3% and 5% for the UCS, ITS and flexural tests. Figure 5 and Table 4 show the compaction curve and optimum values of the mixtures. The content of 5% was used for flexural tests and microscopic and mineralogical analyses.

### 3.3. Unconfined Compressive Strength (UCS)

**Figure 6:**
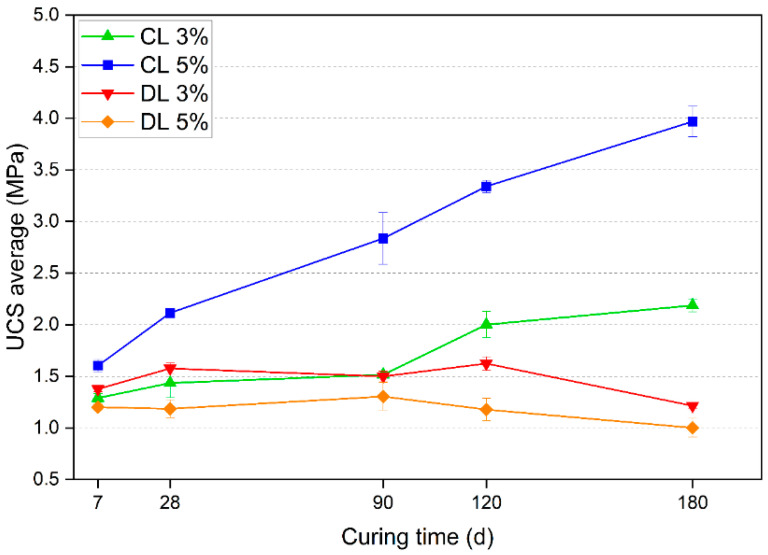
UCS results of the samples with different curing times.

The UCS values for each studied mixture and different curing times are presented in Figure 6. The standard deviations of each triplicate are represented using error bars.

As observed in Figure 6, there is a progressive increase in the UCS as the curing time increases for the CL mixtures. On the other hand, the UCS values of DL mixtures showed no increase with curing time.

**Figure 7:**
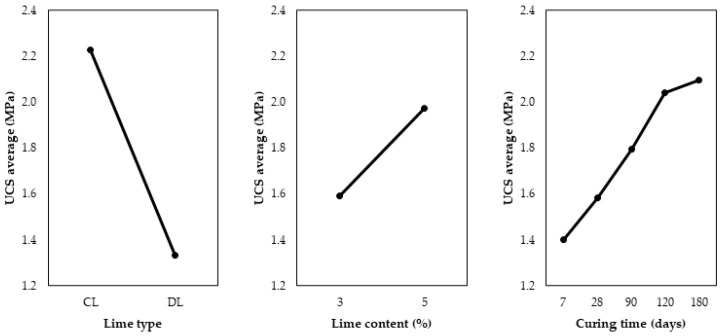
Main effects graph for the UCS of soil-lime samples.

Statistical analyses were performed using Minitab 19 software [30]. Figure 7 shows the main effects of the control factors (curing time, lime type, and lime content) on the UCS. As already obtained by other authors [13,31,32,33], the UCS values are higher as the curing time increases. In addition, the lime content presents higher UCS values when increased from 3 to 5%, as expected, given that the improvement in strength with higher lime content has been consistently observed in previous studies [13,31,34,35]. Finally, the lime type gives higher UCS values when CL is used instead of DL. The superior results obtained with calcitic lime can be attributed to its distinct chemical and physical properties compared to dolomitic lime. Chemically, calcitic lime contains 73% calcium oxide, while dolomitic lime has only 45%. Physically, the finer particle size of calcitic lime provides a larger surface area, enhancing its dissolution rate.

**Figure 8:**
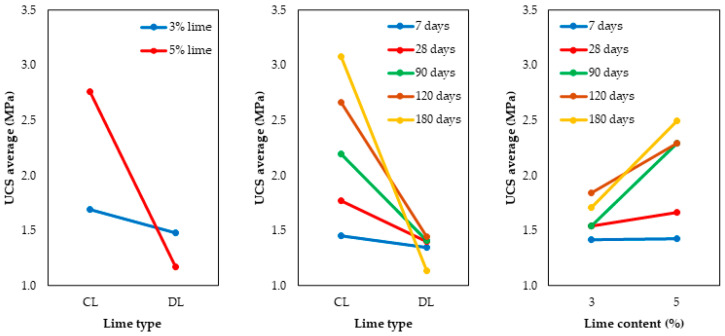
Interaction graphs for the UCS of soil-lime samples.

To observe the second-order interactions between the main effects on the UCS, Figure 8 is presented.

According to the interaction graph, the interaction between the lime type and the lime content impacts UCS differently. For CL, the difference between 3 and 5% is more pronounced. This can be better visualized when the line referring to the 5% content is analyzed separately, which suffers a sudden drop from CL to DL.

Regarding the interaction between the lime type and the curing time, it is noted that CL causes a gradual increase in UCS as the curing time is increased. A different behavior was observed for DL, which reduces UCS values at longer curing times.

Finally, the interaction between the lime content and curing time gradually increases the UCS values when curing time increases. This is also reflected in the rise in the content used when it changes from 3 to 5% for samples of soil with CL.

**Figure 9:**
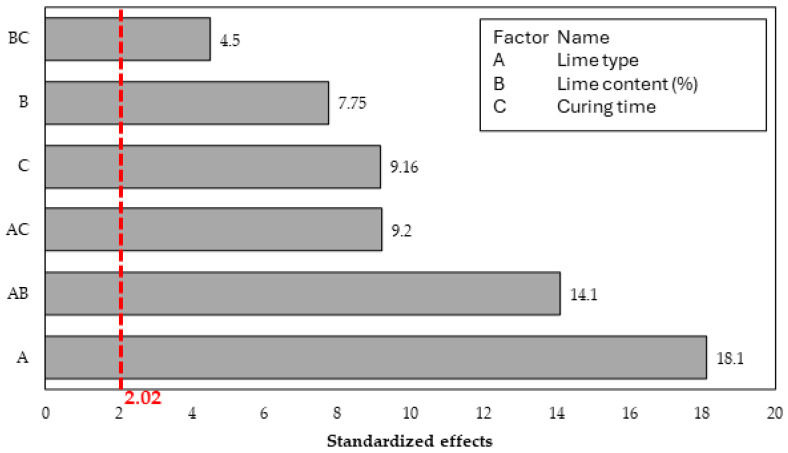
Pareto chart of standardized effects for the UCS of soil-lime samples.

A multiple regression analysis was performed to identify the significant effects and interactions, obtaining a coefficient of determination (R^2^) of 94.6%. Figure 9 presents the Pareto chart of the standardized effects of the mixtures studied. Therefore, the main effects’ magnitude, importance, and interactions were analyzed. The red line represents the reference for the significance of the tested terms at a 0.05 level.

Based on the Pareto chart, it is observed that the control factor with the highest level of significance is the lime type, followed by interaction between the lime type and lime content and the interaction between the lime type and the curing time. Note the relevance of the lime type in the three most significant terms in the Pareto chart.

### 3.4. Indirect Tensile Strength (ITS)

**Figure 10:**
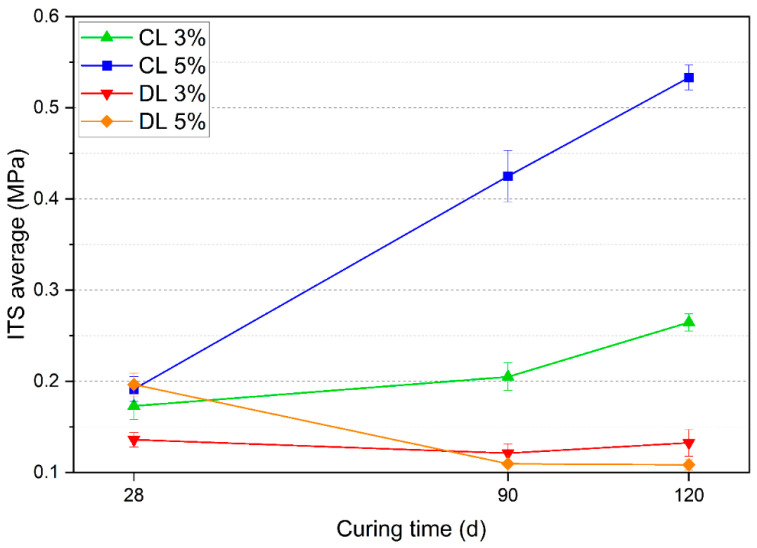
ITS results of the samples with different curing times.

The average ITS values for each mixture are shown in Figure 10. The standard deviations of each triplicate are represented using error bars.

**Figure 11:**
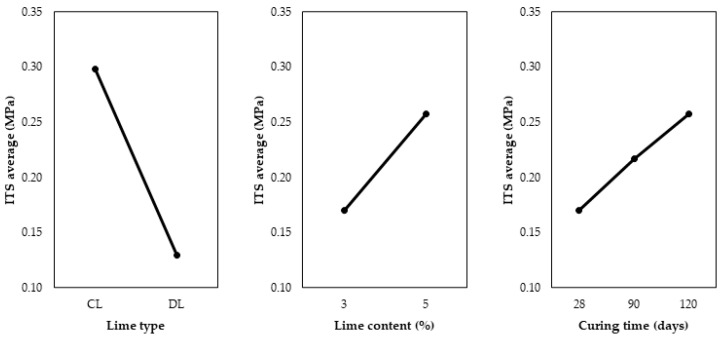
Main effects graph for the ITS of soil-lime samples.

The CL addition resulted in an ITS increase when the curing time was increased, which was more pronounced for the 5% content. For 3% of DL, there was no increase in the ITS with curing time. With a higher amount (5%) of DL, a decrease in ITS values was observed after 28 days. This might be explained by possible carbonation of the mixtures with longer aging. Figure 11 presents the main effects of lime type, lime content, and curing time on the ITS.

**Figure 12:**
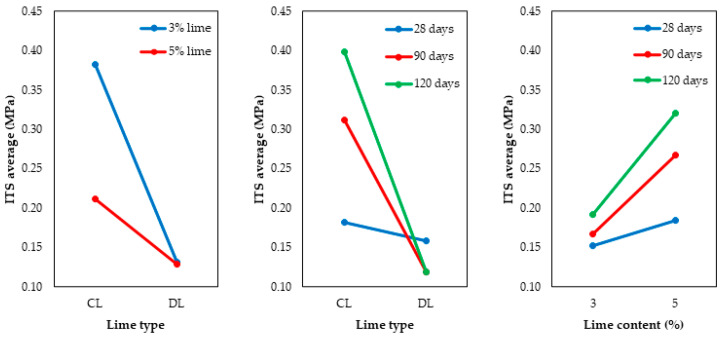
Interaction graphs for ITS of soil-lime samples.

Regarding the lime type, CL promoted higher ITS values than DL, which was the expected result for the same reasons discussed for UCS. The 5% content conferred a higher strength gain than the 3% content. However, this effect was less significant than the lime type. A study conducted by Dhar e Hussain [33] on two soils with lime contents of 3%, 5%, 7%, and 9% demonstrated an increase in ITS with higher lime content. The optimal lime content was 5% for one of the soils and 7% for the other. Considering the three studied curing times (28, 90, and 120 d), there was an increase in the ITS values with the increase in curing time, behavior also observed by Baldovino et al. [13,31]. To observe the second-order interactions between the main effects on the ITS, Figure 12 is presented.

The interaction between the lime type and the curing time had the most significant effect on the ITS, while the interaction between the lime content and the curing time showed a less significant effect. In addition, it is possible to observe that the DL obtained lower strengths than those obtained with CL. The use of 5% content resulted in higher strengths for the CL, but this was not observed for dolomitic lime. Furthermore, for CL mixtures, the effect of curing time on the ITS can be easily visualized, considering the increase in ITS from 28 to 120 d. On the other hand, a strength reduction is observed after 28 d for the DL mixtures.

### 3.5. Flexural Properties

Flexural tests were performed in triplicate with soil mixtures with 5% CL and DL, considering a curing time of 28 d. As mentioned before, the lime content definition was based on the design method, before UCS and ITS results were available, and when it was expected that the higher lime content would lead to higher strengths. The strain at break and static flexural modulus were also determined. Table 5 shows the average values of the parameters obtained from the flexural tests, their respective standard deviations, and coefficients of variation.

The effect of the lime type on flexural strength, strain at break, and static flexural modulus was significant (*p* < 0.05). Regarding the flexural strength and modulus, it can be observed that the CL mixture presented higher values, being stronger and stiffer than the DL mixture. The values of flexural strength are similar to those presented by Mandal et al. [9]. On the other hand, the values of flexural modulus are higher than those reported by Nazari et al. [23]. It is important to note that both studies were carried out using soils from temperate climates.

**Figure 13:**
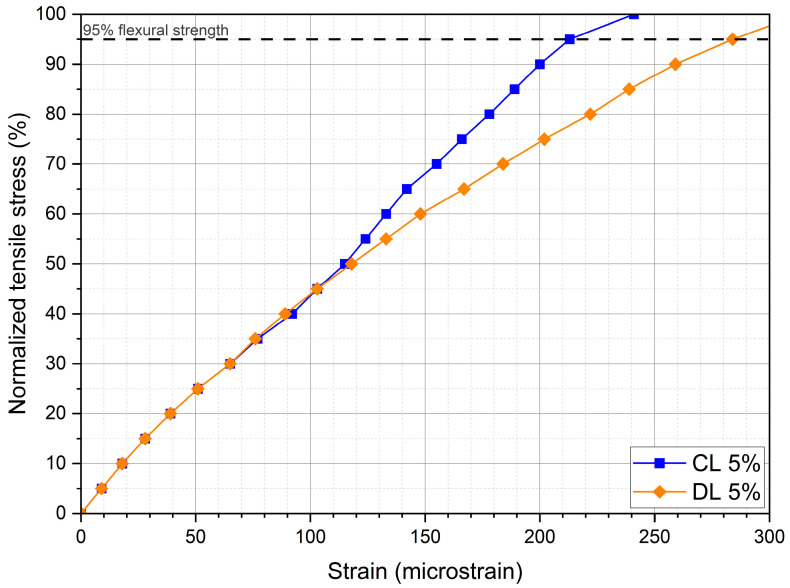
Normalized tensile stress versus tensile strain curves for soil-lime samples.

Figure 13 presents the normalized tensile stresses (%) versus the corresponding tensile strain. An average curve of the tested samples for each mixture was plotted. Considering that the strain at break corresponds to the strain at 95% of the tensile stress, it is observed that the DL mixture presented a higher strain than the CL mixture. Therefore, the mixtures treated with CL showed less flexibility. The values of strain at break are higher than the default values in the South African Mechanistic Pavement Design Method [16], which shows the high flexibility of such mixtures.

### 3.6. Microscopic Analyses

**Figure 14:**
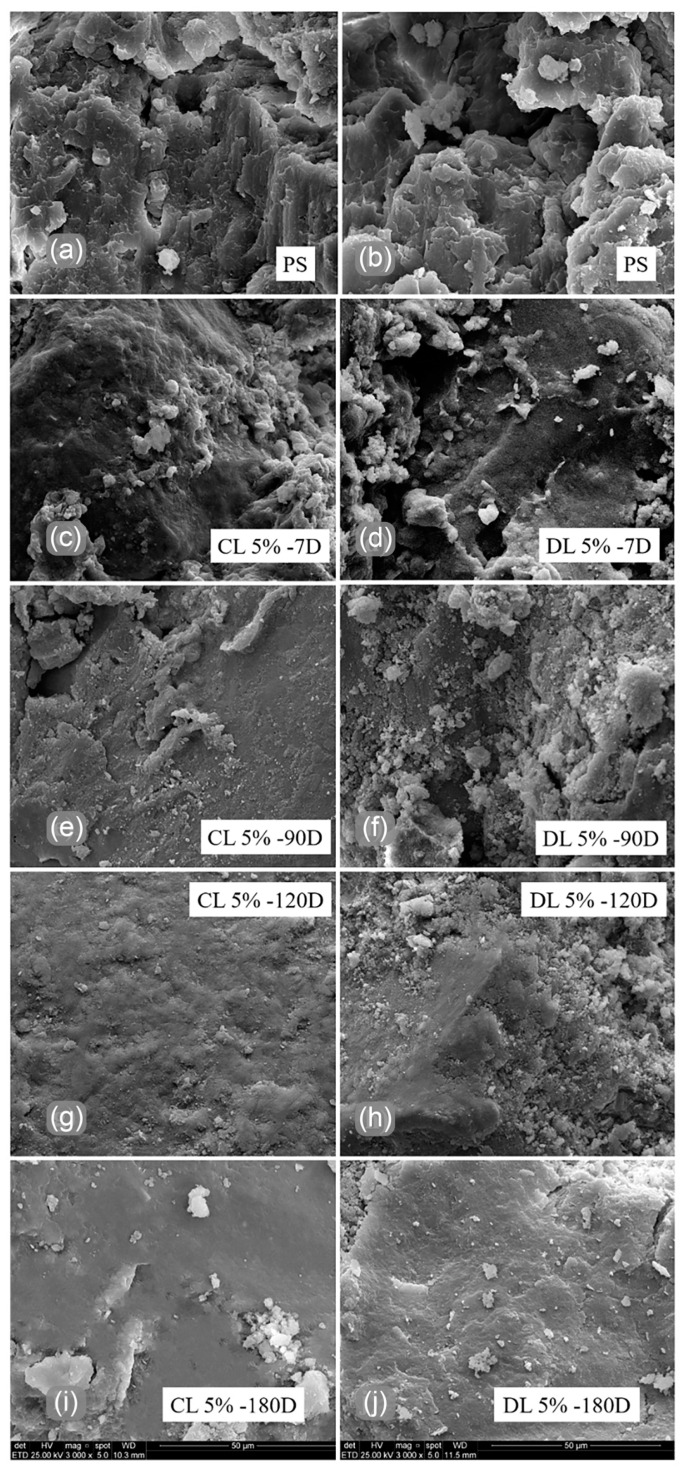
SEM analyses of PS and lime soil blends: (**a**) PS used with CL, (**b**) PS used with DL, (**c**) CL5%-7D, (**d**) DL5%-7D, (**e**) CL5%-90D, (**f**) DL5%-90D, (**g**) CL5%-120D, (**h**) DL5%-120D, (**i**) CL5%-180D, (**j**) DL5%-180D.

Figure 14 presents 3000× magnification SEM images of pure soil and lime soil blends at 7, 90, 120, and 180 days of curing. The textural changes can be evaluated on a microscopic level through these images. Figure 14a,b show that the soil presented high porosity and roughness in the interaggregate matrix. This can be improved with lime addition, as shown in Figure 14c,d.

The blends of soil with CL and DL presented a denser matrix than the pure soil after 7 days of curing. Both Figure 14c,e show flocculation with a dense matrix behind them. Other researchers also observed this through SEM analyses after 24 h of curing [36]. The flocculation presents a flake covered with a gel form which was identified as products derived from primary reactions of lime soil blend. The changes in flocculation particle size at 7 days were attributed to the beginning of the pozzolanic reaction [37,38,39].

At 90 days of curing, no flocculation and a denser matrix were observed for CL and DL. However, in the blend with DL, the flocculation gave way to a dispersed powder on the matrix, as shown in Figure 14e,f. For such an age, the soil particles form a continuous lamellar form interlocking bridge [40]. The CL blends presented a higher interlocking for the following curing ages (120 and 180 days), as shown in Figure 14g,i.

The DL cementing points are presented in Figure 14h,j. The particles lack connection, giving an overlapping sheets aspect, even for greater curing ages. This could be related to weak cementing, explaining the absence of mechanical strength increase. Other authors also observed these reaction products [36], concluding that the soil-lime reactions are non-uniform. The authors indicated that this is probably due to a non-saturation level of the mixtures (below 100%). Therefore, presenting pores partially filled with water promotes non-uniformity in the distribution of cementitious products.

**Figure 15:**
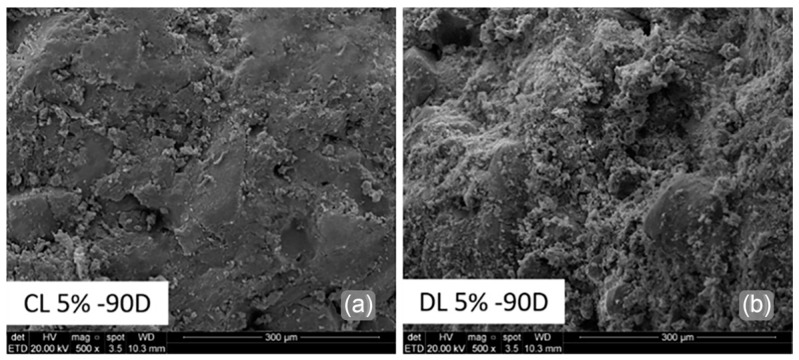
SEM analyses of (**a**) CL 5% and (**b**) DL 5% blends after 90D of curing.

Figure 15 shows a 500× comparison between both blends at 90 days of curing. It is observed that the DL (Figure 15b) presents a dispersed powder on the matrix. This could be related to the high magnesium hydroxide content of DL.

**Figure 16:**
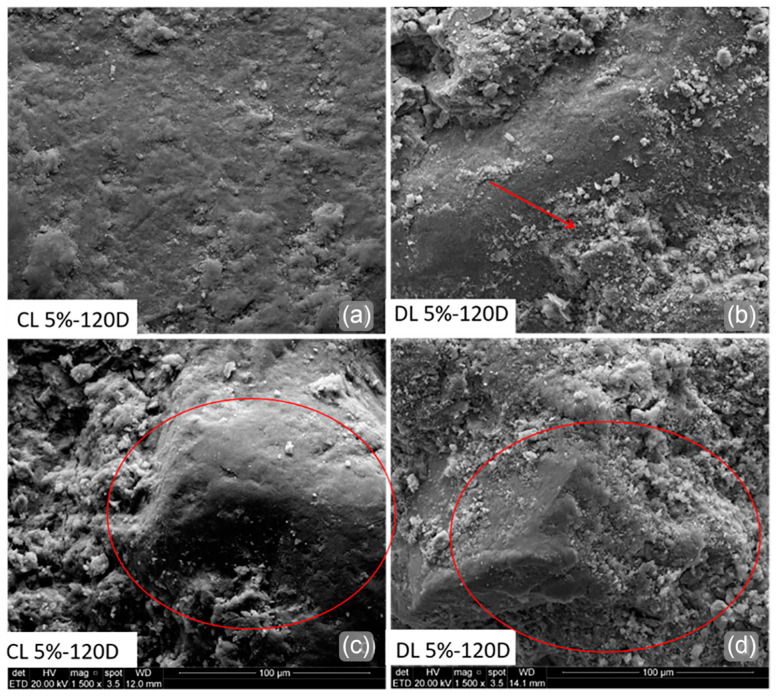
SEM analyses of (**a**,**c**) CL 5% and (**b**,**d**) DL 5% blends after 120D of curing.

Figure 16 illustrates SEM images of CL 5% and DL 5% blends after being cured for 120 days. Both blends presented a denser matrix than pure soil for all curing ages (red circles). The DL blends kept the dispersed powder appearance (Figure 16b—red arrow), while the CL blends presented a matrix density increase. In addition, most DL cementing points showed a fragile characteristic, contributing to a more porous matrix than CL blends. This is also an explanation for low mechanical strength values.

Other authors reported similar trends to those of soil-CL blends in SEM analysis. However, they used soil from temperate climates treated with lime contents varying from 2% to 6% [41]. Furthermore, it has been verified that the strength gain in lime-granular lateritic soil blends is directly related to changes in the soil microstructure resulting from lime reactions [42].

### 3.7. Mineralogical Analyses

**Figure 17:**
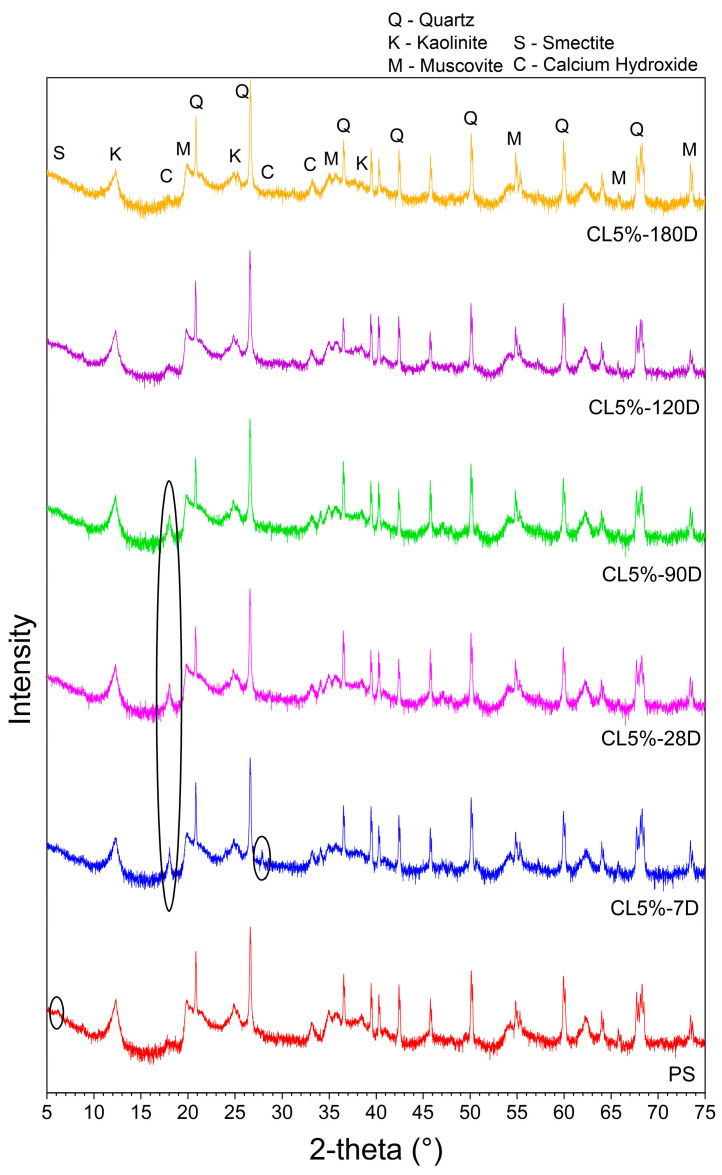
XRD analyses of PS and CL blends for different curing ages (black circles indicate areas of interest).

The XRD analyses of PS compared with CL blends for different curing ages are presented in Figure 17.

The lime and clay minerals consumption were evaluated through XRD analyses performed at the same ages as the UCS tests. According to Figure 17, a reduction in the clay minerals peaks of PS and CL is observed as the curing period increases.

The highest intensity of calcium hydroxide peak was observed at 7 days. This was also observed by Tang et al. [42] when adding lime to a granular lateritic soil, and the intensity of these peaks increased with increasing lime content. Although the lime content effect on the XRD results was not investigated in the present study, the peak intensity decreased with increased curing age. At 120 and 180 days, the calcium hydroxide consumption is almost complete, representing low residual lime content. Furthermore, lime peaks are observed at 2θ 34° for 7, 28, and 90 days of curing but are consumed after 120 days. A peak at 2θ 28° was observed only for 7 days of curing, disappearing at greater curing ages.

Smectite is a clay mineral type 2:1 located at 2θ 6° and was quickly consumed after 7 days of curing in the first reactions between the soil and lime. Other authors also observed this smectite peak reduction after soil treatment with lime [1]. After 7 days, some also reported reduction of clay minerals and development of needle-like structures (C-S-H and C-A-H) that provide strength to the material and guarantee the connection between the clay particles [43].

Kaolinite and muscovite are clay minerals type 1:1 and were consumed as the curing age increased. However, the muscovite consumption was slower when compared to kaolinite.

Unconsumed peaks of hydrated lime were identified at 28 days by Vitale et al. [44] after adding 5% CaO to pure soil. Total consumption was observed after 60 days of curing. According to the authors, the chemical reaction speed after lime addition depends on the mineralogy of clay minerals. Furthermore, low kaolinite reactivity was detected as a delay in new hydrated phases precipitation since the cementing product peaks were well defined only at advanced ages (270 days).

The CL consumption shows the progress of pozzolanic reactions between the lime and clay, as a reduction in intensities of clay minerals peaks was observed for greater curing ages. Even though no C-S-H and C-A-H peaks were reported, low quantities of these compounds may be present since the increase in mechanical strength and textural changes were observed in UCS tests and SEM, respectively.

**Figure 18:**
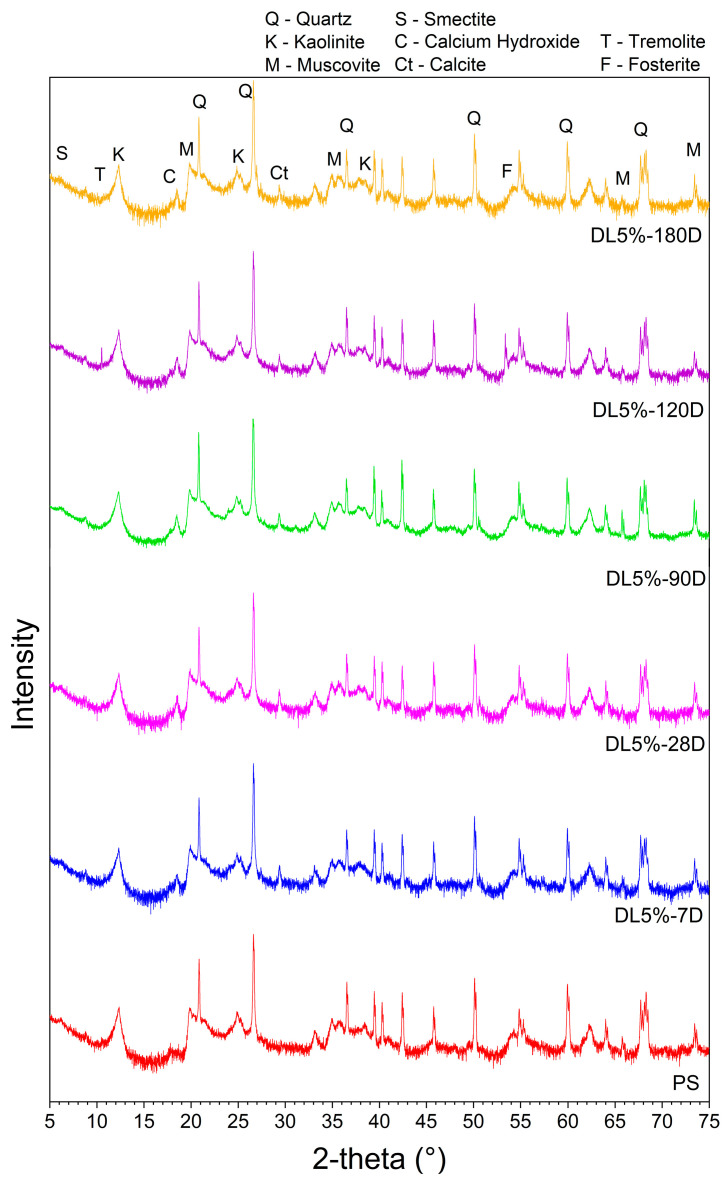
XRD analyses of PS and DL blends for different curing ages.

The XRD analyses of PS compared with DL blends for different curing ages are presented in Figure 18.

According to Figure 18, it can be seen that smectite is partially consumed after 7 days. The lime addition promoted a peak of calcium hydroxide at 2θ 19° and 28° at 7 days. The former kept its intensity until 180 days, and the latter was consumed at 28 days of curing. The kaolinite and muscovite peaks were reduced as the curing time increased. The delay in clay minerals consumption of DL in comparison with CL was also observed in other studies of a Red-Yellow Argisol [45].

A calcite (calcium carbonate) peak was observed at 2θ 30° from 7 days. In addition, tremolite and forsterite peaks were identified from 90 days, at 2θ 11° and 54°, respectively. These compounds agree with other presented results since a significant textural change was observed in SEM, and no increase in UCS values was observed even for greater curing ages. Additionally, clay minerals consumption occurs until 180 days.

Although it was not evaluated in the present study, previous research using XRD tests has shown that that at higher lime content, expansion of the cementitious compounds occurs, leading to a weak structure and decreasing strength [46]. This might explain why the mixtures with 3% DL were stronger than those with 5% DL (UCS and ITS results).

## 4. Conclusions

The results led to the conclusion that the controllable factors (lime type, lime content, and curing time) significantly affect the mechanical, microstructural, and mineralogical behavior of the tropical soil characterized as a Red Argisol treated with lime. The type of lime is the controllable factor that presented the highest significance level, so calcitic lime (CL) provided higher strength and stiffness than dolomitic lime (DL). The improvement in mechanical behavior is evidenced by mineralogical analyses, which showed higher consumption of soil clay minerals and calcium hydroxide, and by microstructural analyses, in which the CL mixtures presented a more closed matrix than the DL mixtures. For the latter, it was observed that there is no uniformity in the distribution of cementing points generated, indicating the formation of weak cementation.

The higher calcium oxide (CaO) content of CL compared to DL demonstrates greater availability for developing pozzolanic reactions, which explains the better results obtained with CL. This characteristic also affected the development of reactions with the increase in curing time; a gradual increase in strength was observed when CL was added. Another point to note is that the influence of the lime content, evaluated in the unconfined compressive strength (UCS) and indirect tensile strength (ITS) tests, was also significant only for CL, in which adding 5% provided greater strength than 3%. The flexural properties, including the strain at break, essential for pavement design and rarely reported for such mixtures, were assessed, and showed that the mixtures with DL are more flexible than those with CL.

Overall, it is concluded that lime stabilization is a great alternative to improve the mechanical behavior of the studied tropical soil for pavement applications, showing that the technique could be reproduced for other tropical soils. The technique would enhance local soil engineering properties while also being sustainable by reducing the need for raw material extraction/transportation and improving resilience to climate-related issues such as floods.

## Figures and Tables

**Table 1:** XFR analysis of PS.

Oxide	% Mass
MgO	8.854
Al_2_O_3_	25.833
SiO_2_	48.975
K_2_O	1.025
CaO	0.098
TiO_2_	0.833
MnO	0.008
Fe_2_O_3_	6.017
SrO	0.006
ZrO_2_	0.045
LOI	2.430

**Table 2:** Chemical analysis of PS.

Parameter	Soil
Clay (%)	>60
pH (H_2_O)	4.4
SMP index	5.1
P (mg·dm^−3^)	1.1
K (mg·dm^−3^)	25.0
Organic matter (%)	0.6
Exchangeable Al (cmolc/dm^3^)	3.0
Exchangeable Ca (cmolc/dm^3^)	1.0
Al + H (cmolc/dm^3^)	12.3
CEC (cmolc/dm^3^)	14.2
Base saturation (%)	14.0
Aluminum saturation (%)	60.3
Ca/Mg ratio	1.1
Ca/K ratio	16.0
Mg/K ratio	14.0
S (cmolc/dm^3^)	0.3
Zn (cmolc/dm^3^)	0.3
Cu (cmolc/dm^3^)	0.6
B (cmolc/dm^3^)	0.3
Mn (cmolc/dm^3^)	7.0

**Table 3:** Characterization of the limes.

Parameter	Calcitic	Dolomitic
CaO_E_ (%)	73.08	45.22
MgO (%)	0.55	31.10
Ca(OH)_2_ (%)	92.63	44.42
Mg(OH)_2_ (%)	0.80	39.63
(CaO + MgO)_E_ (%)	98.15	93.06
Al_2_O_3_ (%)	0.05	0.41
SiO_2_ (%)	0.12	3.02
Fe_2_O_3_ (%)	0.18	1.29
CO_2_ (%)	2.42	2.88
S (%)	0.03	0.01
Na_2_O (%)	-	0.11
K_2_O (%)	-	0.38
P_2_O_5_ (%)	0.06	0.03
SrO (%)	0.39	0.03
ZrO_2_ (%)	0.05	0.01
MnO (%)	0.05	0.01
ZnO (%)	-	0.09
TiO_2_ (%)	-	0.05
BaO (%)	-	0.03
Cl (%)	0.02	0.01
LOI (%)	25.22	17.99
Humidity (%)	0.70	0.40
Specific mass (g·cm^−3^)	0.40	0.61
Retained on the #28 sieve (0.60 mm)	0	0
Retained on the #200 sieve (0.075 mm)	0.21	17.79
E: exchangeable		

**Table 4:** Optimum parameters of compaction curves.

Sample	Code	ω_optimum_ (%)	ρ_d_ (g·cm^−3^)
Pure soil	PS	17.90	1.875
Soil + 3% calcitic lime	CL 3%	18.95	1.838
Soil + 3% calcitic lime	CL 5%	18.90	1.822
Soil + 5% dolomitic lime	DL 3%	18.10	1.856
Soil + 5% dolomitic lime	DL 5%	19.60	1.844

**Table 5:** Flexural test results for the soil-lime samples.

	CL 5%			DL 5%		
	$\bar{x}$	σ	CV (%)	$\bar{x}$	σ	CV (%)
Flexural strength (MPa)	0.37	0.01	3.80	0.22	0.02	9.90
Strain at break (microstrain)	214.00	3.54	1.70	283.00	14.14	5.00
Static flexural modulus (MPa)	1617.00	103.94	6.40	1013.00	5.66	0.60

## Data Availability

The original contributions presented in the study are included in the article, further inquiries can be directed to the corresponding author.

## Abbreviations

AASHTO	American Association of State Highway and Transportation Officials
ABNT	Associação Brasileira de Normas Técnicas
ASTM	American Society for Testing and Materials
CEC	Cation Exchange Capacity
CH	Inorganic clay of high compressibility
CL	Calcitic lime
DNIT	Departamento Nacional de Infraestrutura
DL	Dolomitic lime
EMBRAPA	Brazilian Agricultural Research Corporation
ITS	Indirect Tensile Strength
NCHRP	National Cooperative Highway Research Program
PS	Pure Soil
UCS	Unconfined Compressive Strength
USCS	Unified Soil Classification System
SEM	Scanning Electron Microscopy
XRD	X-Ray Diffraction
XRF	X-Ray Fluorescence
